# Supplementary material for: FAK Inhibitor-Based Combinations with MEK or PKC Inhibitors Trigger Synergistic Antitumor Effects in Uveal Melanoma
Source: Cancers (Basel). 2023 Apr 13;15(8):2280. doi: 10.3390/cancers15082280 (PMC10136875; doi:10.3390/cancers15082280)
Supplement: Supplementary file 1 [file cancers-15-02280-s001.zip › REV Supp_Figures.pptx]

## Slide 1
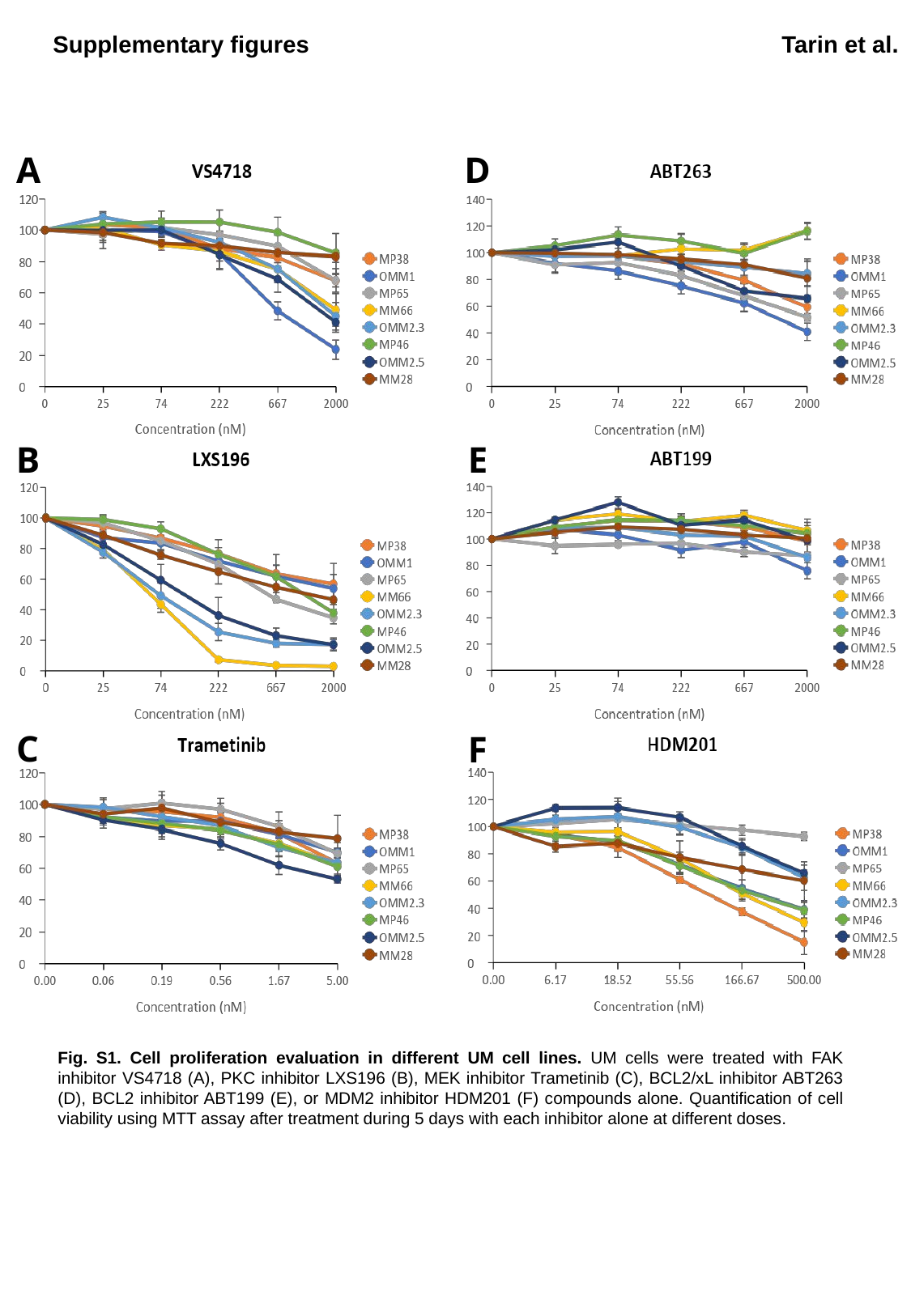

Supplementary figures				Tarin et al.
A
D
B
E
C
F
Fig. S1. Cell proliferation evaluation in different UM cell lines. UM cells were treated with FAK inhibitor VS4718 (A), PKC inhibitor LXS196 (B), MEK inhibitor Trametinib (C), BCL2/xL inhibitor ABT263 (D), BCL2 inhibitor ABT199 (E), or MDM2 inhibitor HDM201 (F) compounds alone. Quantification of cell viability using MTT assay after treatment during 5 days with each inhibitor alone at different doses.

## Slide 2
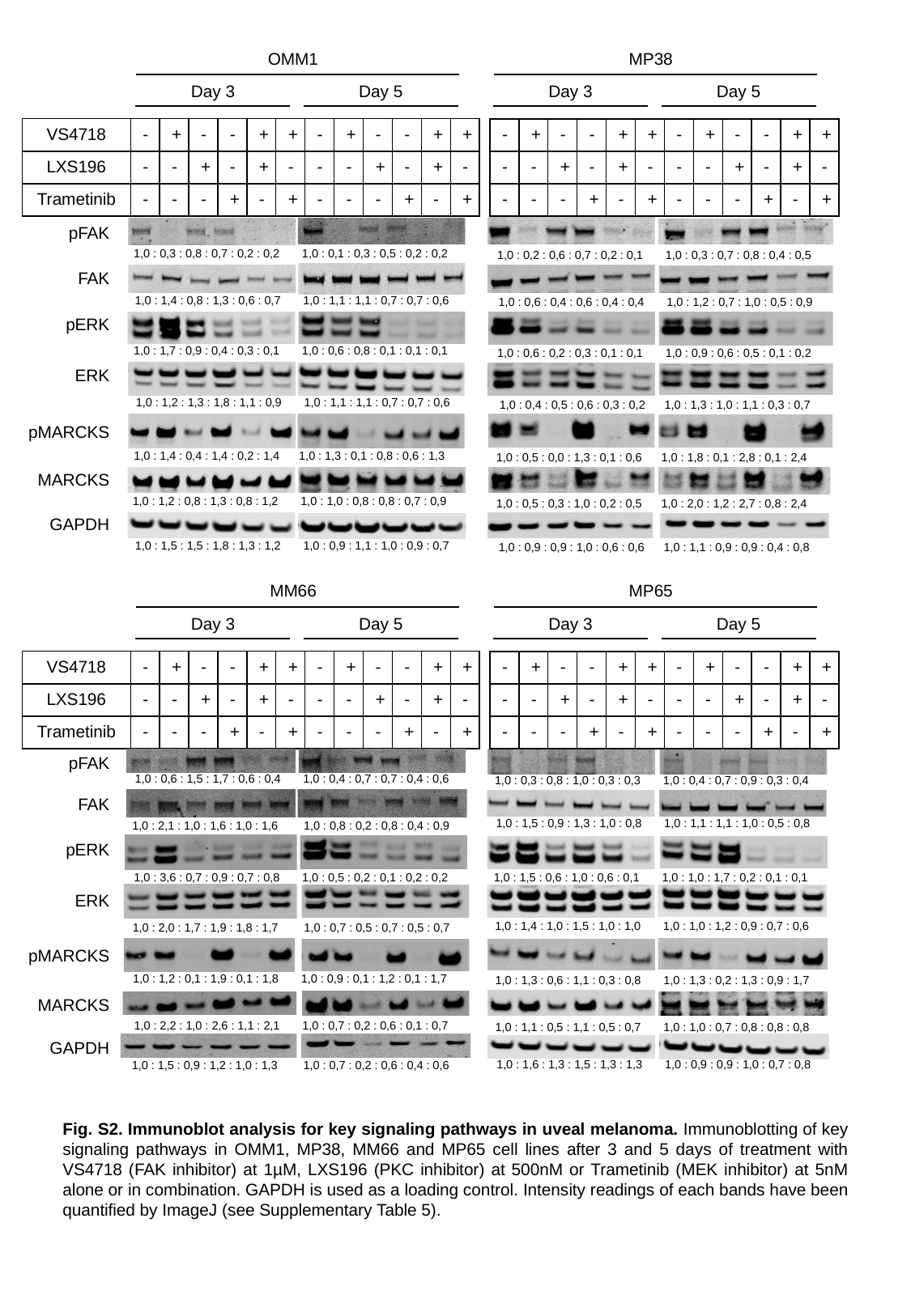

OMM1
MP38
Day 3 	 Day 5
Day 3	 Day 5
| VS4718 | - | + | - | - | + | + | - | + | - | - | + | + |
| --- | --- | --- | --- | --- | --- | --- | --- | --- | --- | --- | --- | --- |
| LXS196 | - | - | + | - | + | - | - | - | + | - | + | - |
| Trametinib | - | - | - | + | - | + | - | - | - | + | - | + |
| - | + | - | - | + | + | - | + | - | - | + | + |
| --- | --- | --- | --- | --- | --- | --- | --- | --- | --- | --- | --- |
| - | - | + | - | + | - | - | - | + | - | + | - |
| - | - | - | + | - | + | - | - | - | + | - | + |
pFAK
1,0 : 0,3 : 0,8 : 0,7 : 0,2 : 0,2 1,0 : 0,1 : 0,3 : 0,5 : 0,2 : 0,2
1,0 : 0,2 : 0,6 : 0,7 : 0,2 : 0,1 1,0 : 0,3 : 0,7 : 0,8 : 0,4 : 0,5
FAK
1,0 : 1,4 : 0,8 : 1,3 : 0,6 : 0,7 1,0 : 1,1 : 1,1 : 0,7 : 0,7 : 0,6
1,0 : 0,6 : 0,4 : 0,6 : 0,4 : 0,4 1,0 : 1,2 : 0,7 : 1,0 : 0,5 : 0,9
pERK
1,0 : 1,7 : 0,9 : 0,4 : 0,3 : 0,1 1,0 : 0,6 : 0,8 : 0,1 : 0,1 : 0,1
1,0 : 0,6 : 0,2 : 0,3 : 0,1 : 0,1 1,0 : 0,9 : 0,6 : 0,5 : 0,1 : 0,2
ERK
1,0 : 1,2 : 1,3 : 1,8 : 1,1 : 0,9 1,0 : 1,1 : 1,1 : 0,7 : 0,7 : 0,6
1,0 : 0,4 : 0,5 : 0,6 : 0,3 : 0,2 1,0 : 1,3 : 1,0 : 1,1 : 0,3 : 0,7
pMARCKS
1,0 : 1,4 : 0,4 : 1,4 : 0,2 : 1,4 1,0 : 1,3 : 0,1 : 0,8 : 0,6 : 1,3
1,0 : 0,5 : 0,0 : 1,3 : 0,1 : 0,6 1,0 : 1,8 : 0,1 : 2,8 : 0,1 : 2,4
MARCKS
1,0 : 1,2 : 0,8 : 1,3 : 0,8 : 1,2 1,0 : 1,0 : 0,8 : 0,8 : 0,7 : 0,9
1,0 : 0,5 : 0,3 : 1,0 : 0,2 : 0,5 1,0 : 2,0 : 1,2 : 2,7 : 0,8 : 2,4
GAPDH
1,0 : 1,5 : 1,5 : 1,8 : 1,3 : 1,2 1,0 : 0,9 : 1,1 : 1,0 : 0,9 : 0,7
1,0 : 0,9 : 0,9 : 1,0 : 0,6 : 0,6 1,0 : 1,1 : 0,9 : 0,9 : 0,4 : 0,8
MM66
MP65
Day 3	 Day 5
Day 3	 Day 5
| VS4718 | - | + | - | - | + | + | - | + | - | - | + | + |
| --- | --- | --- | --- | --- | --- | --- | --- | --- | --- | --- | --- | --- |
| LXS196 | - | - | + | - | + | - | - | - | + | - | + | - |
| Trametinib | - | - | - | + | - | + | - | - | - | + | - | + |
| - | + | - | - | + | + | - | + | - | - | + | + |
| --- | --- | --- | --- | --- | --- | --- | --- | --- | --- | --- | --- |
| - | - | + | - | + | - | - | - | + | - | + | - |
| - | - | - | + | - | + | - | - | - | + | - | + |
pFAK
1,0 : 0,6 : 1,5 : 1,7 : 0,6 : 0,4 1,0 : 0,4 : 0,7 : 0,7 : 0,4 : 0,6
1,0 : 0,3 : 0,8 : 1,0 : 0,3 : 0,3 1,0 : 0,4 : 0,7 : 0,9 : 0,3 : 0,4
FAK
1,0 : 1,5 : 0,9 : 1,3 : 1,0 : 0,8 1,0 : 1,1 : 1,1 : 1,0 : 0,5 : 0,8
1,0 : 2,1 : 1,0 : 1,6 : 1,0 : 1,6 1,0 : 0,8 : 0,2 : 0,8 : 0,4 : 0,9
pERK
1,0 : 3,6 : 0,7 : 0,9 : 0,7 : 0,8 1,0 : 0,5 : 0,2 : 0,1 : 0,2 : 0,2
1,0 : 1,5 : 0,6 : 1,0 : 0,6 : 0,1 1,0 : 1,0 : 1,7 : 0,2 : 0,1 : 0,1
ERK
1,0 : 1,4 : 1,0 : 1,5 : 1,0 : 1,0 1,0 : 1,0 : 1,2 : 0,9 : 0,7 : 0,6
1,0 : 2,0 : 1,7 : 1,9 : 1,8 : 1,7 1,0 : 0,7 : 0,5 : 0,7 : 0,5 : 0,7
pMARCKS
1,0 : 1,2 : 0,1 : 1,9 : 0,1 : 1,8 1,0 : 0,9 : 0,1 : 1,2 : 0,1 : 1,7
1,0 : 1,3 : 0,6 : 1,1 : 0,3 : 0,8 1,0 : 1,3 : 0,2 : 1,3 : 0,9 : 1,7
MARCKS
1,0 : 2,2 : 1,0 : 2,6 : 1,1 : 2,1 1,0 : 0,7 : 0,2 : 0,6 : 0,1 : 0,7
1,0 : 1,1 : 0,5 : 1,1 : 0,5 : 0,7 1,0 : 1,0 : 0,7 : 0,8 : 0,8 : 0,8
GAPDH
1,0 : 1,6 : 1,3 : 1,5 : 1,3 : 1,3 1,0 : 0,9 : 0,9 : 1,0 : 0,7 : 0,8
1,0 : 1,5 : 0,9 : 1,2 : 1,0 : 1,3 1,0 : 0,7 : 0,2 : 0,6 : 0,4 : 0,6
Fig. S2. Immunoblot analysis for key signaling pathways in uveal melanoma. Immunoblotting of key signaling pathways in OMM1, MP38, MM66 and MP65 cell lines after 3 and 5 days of treatment with VS4718 (FAK inhibitor) at 1µM, LXS196 (PKC inhibitor) at 500nM or Trametinib (MEK inhibitor) at 5nM alone or in combination. GAPDH is used as a loading control. Intensity readings of each bands have been quantified by ImageJ (see Supplementary Table 5).

## Slide 3
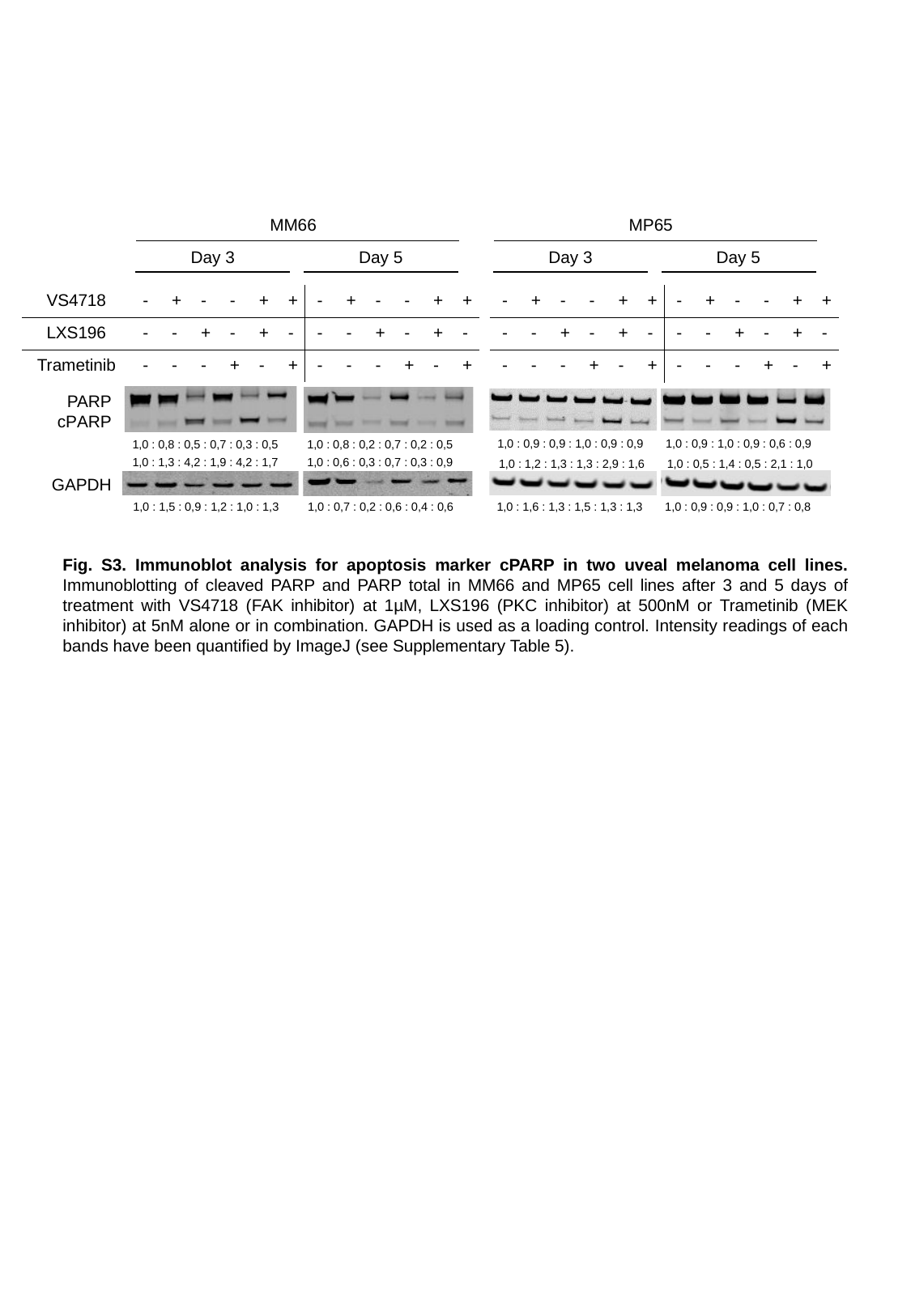

MM66
MP65
Day 3	 Day 5
Day 3	 Day 5
| VS4718 | - | + | - | - | + | + | - | + | - | - | + | + |
| --- | --- | --- | --- | --- | --- | --- | --- | --- | --- | --- | --- | --- |
| LXS196 | - | - | + | - | + | - | - | - | + | - | + | - |
| Trametinib | - | - | - | + | - | + | - | - | - | + | - | + |
| - | + | - | - | + | + | - | + | - | - | + | + |
| --- | --- | --- | --- | --- | --- | --- | --- | --- | --- | --- | --- |
| - | - | + | - | + | - | - | - | + | - | + | - |
| - | - | - | + | - | + | - | - | - | + | - | + |
PARP
cPARP
1,0 : 0,9 : 0,9 : 1,0 : 0,9 : 0,9 1,0 : 0,9 : 1,0 : 0,9 : 0,6 : 0,9
1,0 : 0,8 : 0,5 : 0,7 : 0,3 : 0,5 1,0 : 0,8 : 0,2 : 0,7 : 0,2 : 0,5
1,0 : 1,3 : 4,2 : 1,9 : 4,2 : 1,7 1,0 : 0,6 : 0,3 : 0,7 : 0,3 : 0,9
1,0 : 1,2 : 1,3 : 1,3 : 2,9 : 1,6 1,0 : 0,5 : 1,4 : 0,5 : 2,1 : 1,0
GAPDH
1,0 : 1,5 : 0,9 : 1,2 : 1,0 : 1,3 1,0 : 0,7 : 0,2 : 0,6 : 0,4 : 0,6
1,0 : 1,6 : 1,3 : 1,5 : 1,3 : 1,3 1,0 : 0,9 : 0,9 : 1,0 : 0,7 : 0,8
Fig. S3. Immunoblot analysis for apoptosis marker cPARP in two uveal melanoma cell lines. Immunoblotting of cleaved PARP and PARP total in MM66 and MP65 cell lines after 3 and 5 days of treatment with VS4718 (FAK inhibitor) at 1µM, LXS196 (PKC inhibitor) at 500nM or Trametinib (MEK inhibitor) at 5nM alone or in combination. GAPDH is used as a loading control. Intensity readings of each bands have been quantified by ImageJ (see Supplementary Table 5).

## Slide 4
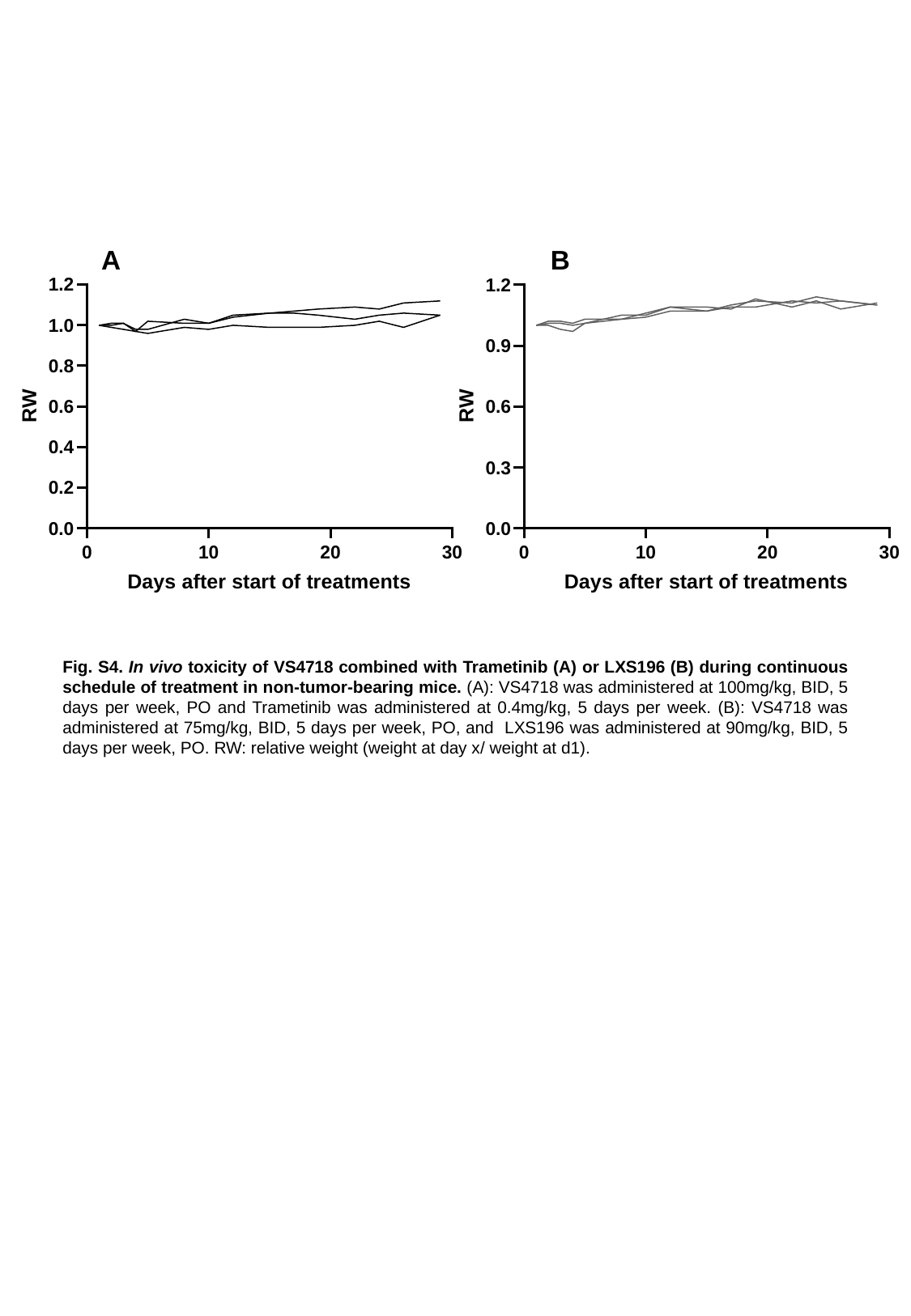

A
B
Fig. S4. In vivo toxicity of VS4718 combined with Trametinib (A) or LXS196 (B) during continuous schedule of treatment in non-tumor-bearing mice. (A): VS4718 was administered at 100mg/kg, BID, 5 days per week, PO and Trametinib was administered at 0.4mg/kg, 5 days per week. (B): VS4718 was administered at 75mg/kg, BID, 5 days per week, PO, and LXS196 was administered at 90mg/kg, BID, 5 days per week, PO. RW: relative weight (weight at day x/ weight at d1).

## Slide 5
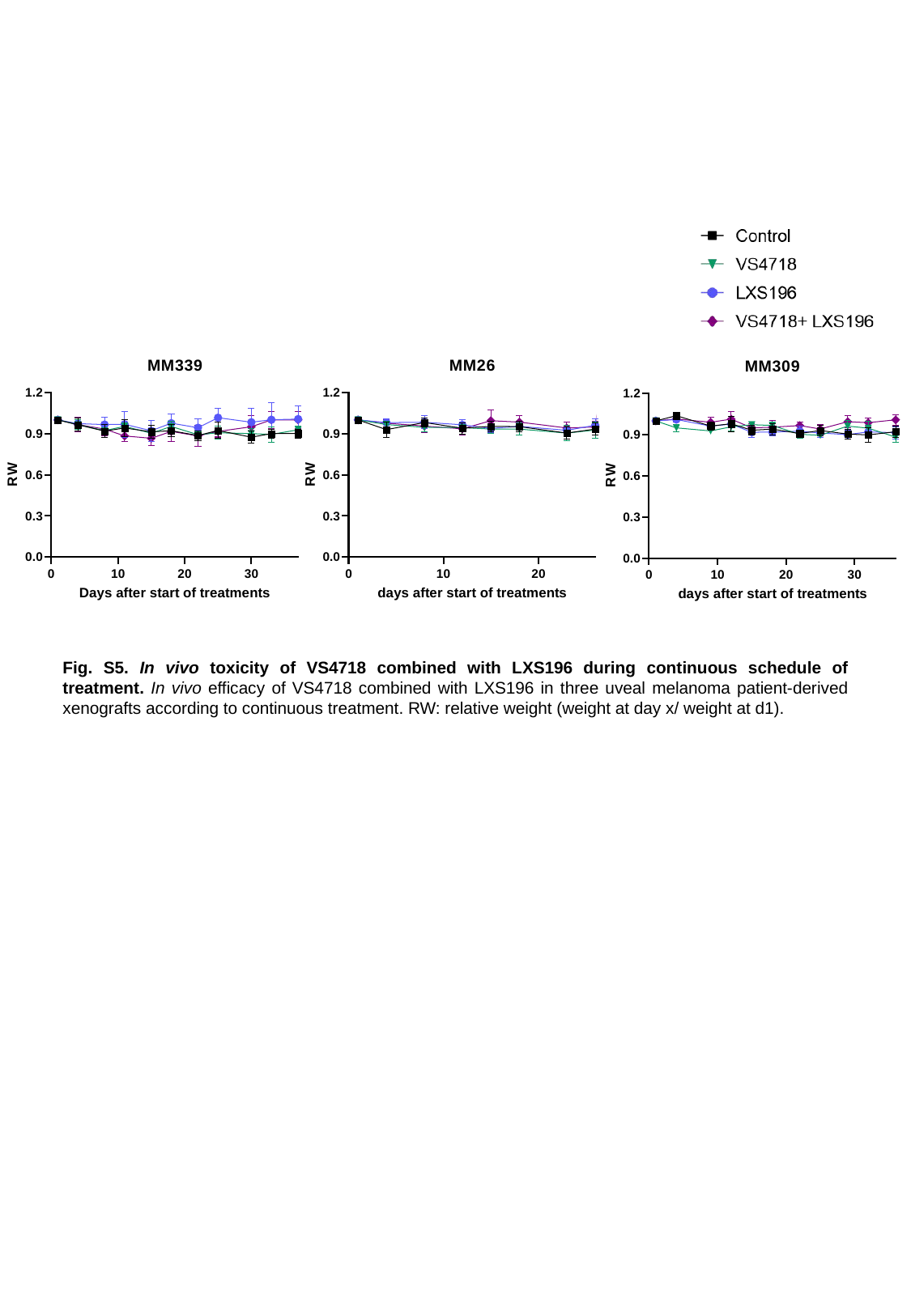

Fig. S5. In vivo toxicity of VS4718 combined with LXS196 during continuous schedule of treatment. In vivo efficacy of VS4718 combined with LXS196 in three uveal melanoma patient-derived xenografts according to continuous treatment. RW: relative weight (weight at day x/ weight at d1).

## Slide 6
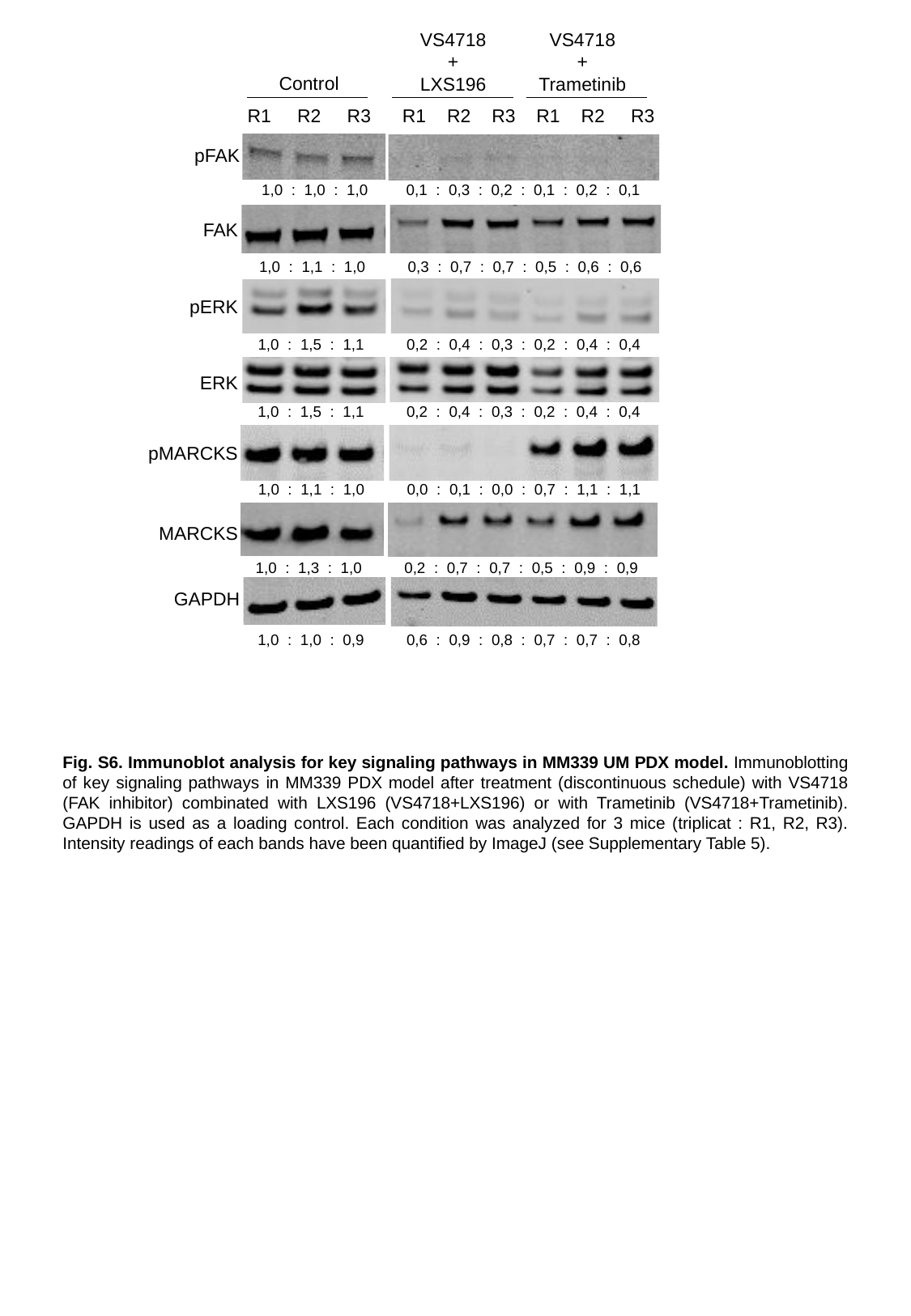

VS4718
+
LXS196
VS4718
+
Trametinib
Control
R1 R2 R3 R1 R2 R3 R1 R2 R3
pFAK
1,0 : 1,0 : 1,0 0,1 : 0,3 : 0,2 : 0,1 : 0,2 : 0,1
FAK
1,0 : 1,1 : 1,0 0,3 : 0,7 : 0,7 : 0,5 : 0,6 : 0,6
pERK
1,0 : 1,5 : 1,1 0,2 : 0,4 : 0,3 : 0,2 : 0,4 : 0,4
ERK
1,0 : 1,5 : 1,1 0,2 : 0,4 : 0,3 : 0,2 : 0,4 : 0,4
pMARCKS
1,0 : 1,1 : 1,0 0,0 : 0,1 : 0,0 : 0,7 : 1,1 : 1,1
MARCKS
1,0 : 1,3 : 1,0 0,2 : 0,7 : 0,7 : 0,5 : 0,9 : 0,9
GAPDH
1,0 : 1,0 : 0,9 0,6 : 0,9 : 0,8 : 0,7 : 0,7 : 0,8
Fig. S6. Immunoblot analysis for key signaling pathways in MM339 UM PDX model. Immunoblotting of key signaling pathways in MM339 PDX model after treatment (discontinuous schedule) with VS4718 (FAK inhibitor) combinated with LXS196 (VS4718+LXS196) or with Trametinib (VS4718+Trametinib). GAPDH is used as a loading control. Each condition was analyzed for 3 mice (triplicat : R1, R2, R3). Intensity readings of each bands have been quantified by ImageJ (see Supplementary Table 5).

## Slide 7
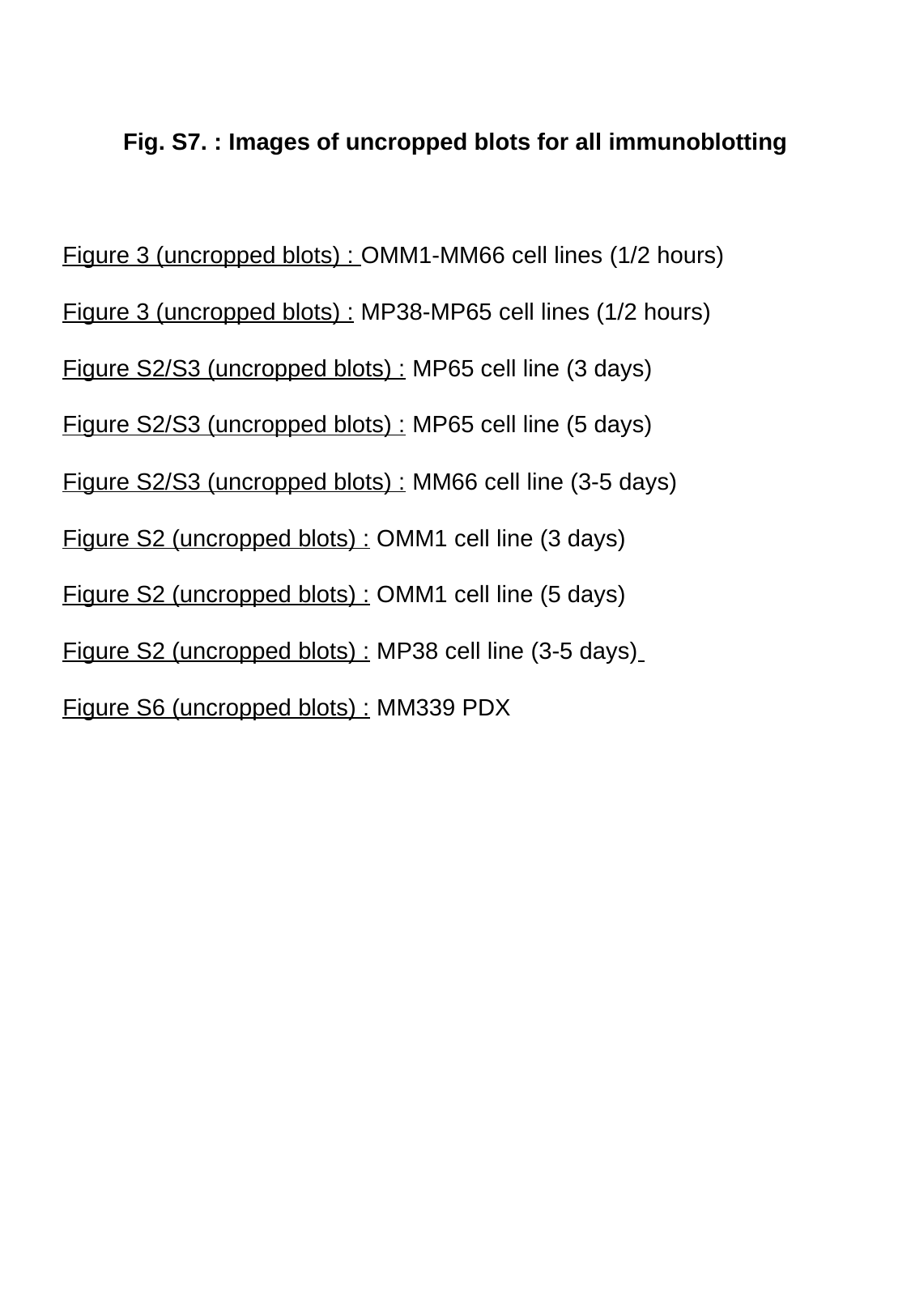

Fig. S7. : Images of uncropped blots for all immunoblotting
Figure 3 (uncropped blots) : OMM1-MM66 cell lines (1/2 hours)
Figure 3 (uncropped blots) : MP38-MP65 cell lines (1/2 hours)
Figure S2/S3 (uncropped blots) : MP65 cell line (3 days)
Figure S2/S3 (uncropped blots) : MP65 cell line (5 days)
Figure S2/S3 (uncropped blots) : MM66 cell line (3-5 days)
Figure S2 (uncropped blots) : OMM1 cell line (3 days)
Figure S2 (uncropped blots) : OMM1 cell line (5 days)
Figure S2 (uncropped blots) : MP38 cell line (3-5 days)
Figure S6 (uncropped blots) : MM339 PDX

## Slide 8
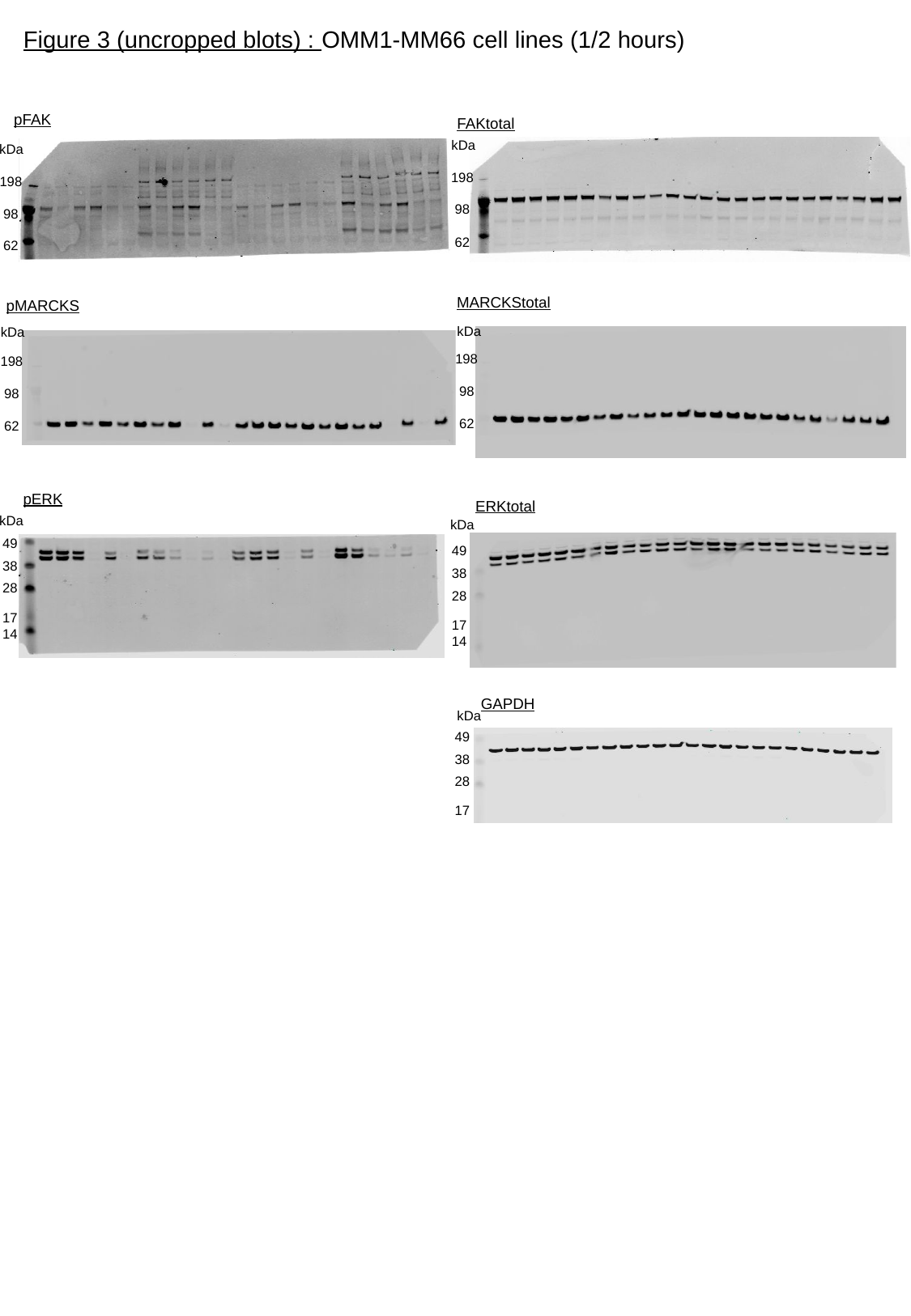

Figure 3 (uncropped blots) : OMM1-MM66 cell lines (1/2 hours)
pFAK
FAKtotal
kDa
kDa
198
98
62
198
98
62
MARCKStotal
pMARCKS
kDa
kDa
198
98
62
198
98
62
pERK
ERKtotal
kDa
kDa
49
38
28
17
14
49
38
28
17
14
GAPDH
kDa
49
38
28
17

## Slide 9
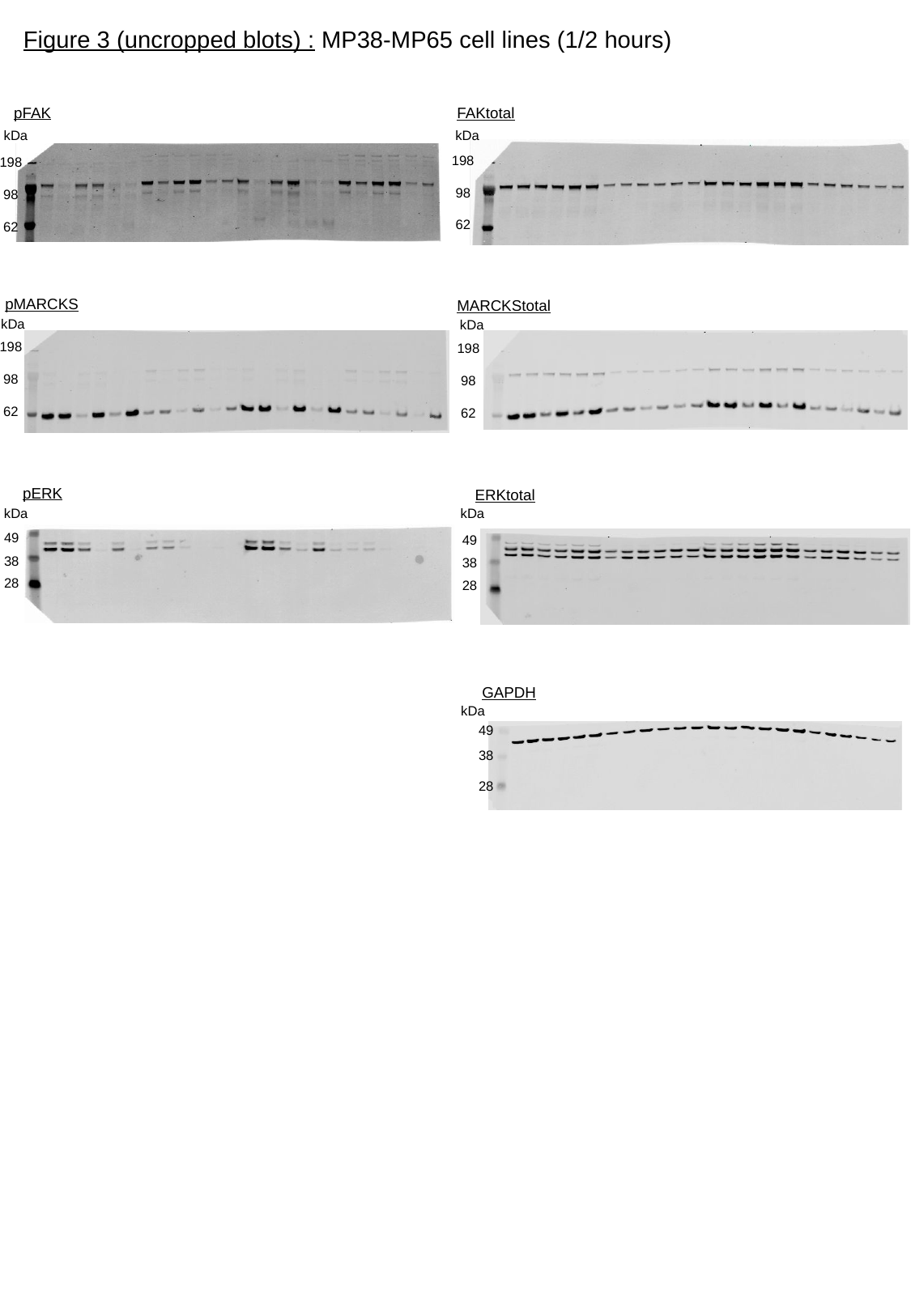

Figure 3 (uncropped blots) : MP38-MP65 cell lines (1/2 hours)
pFAK
FAKtotal
kDa
kDa
198
98
62
198
98
62
pMARCKS
MARCKStotal
kDa
kDa
198
98
62
198
98
62
pERK
ERKtotal
kDa
kDa
49
38
28
49
38
28
GAPDH
kDa
49
38
28

## Slide 10
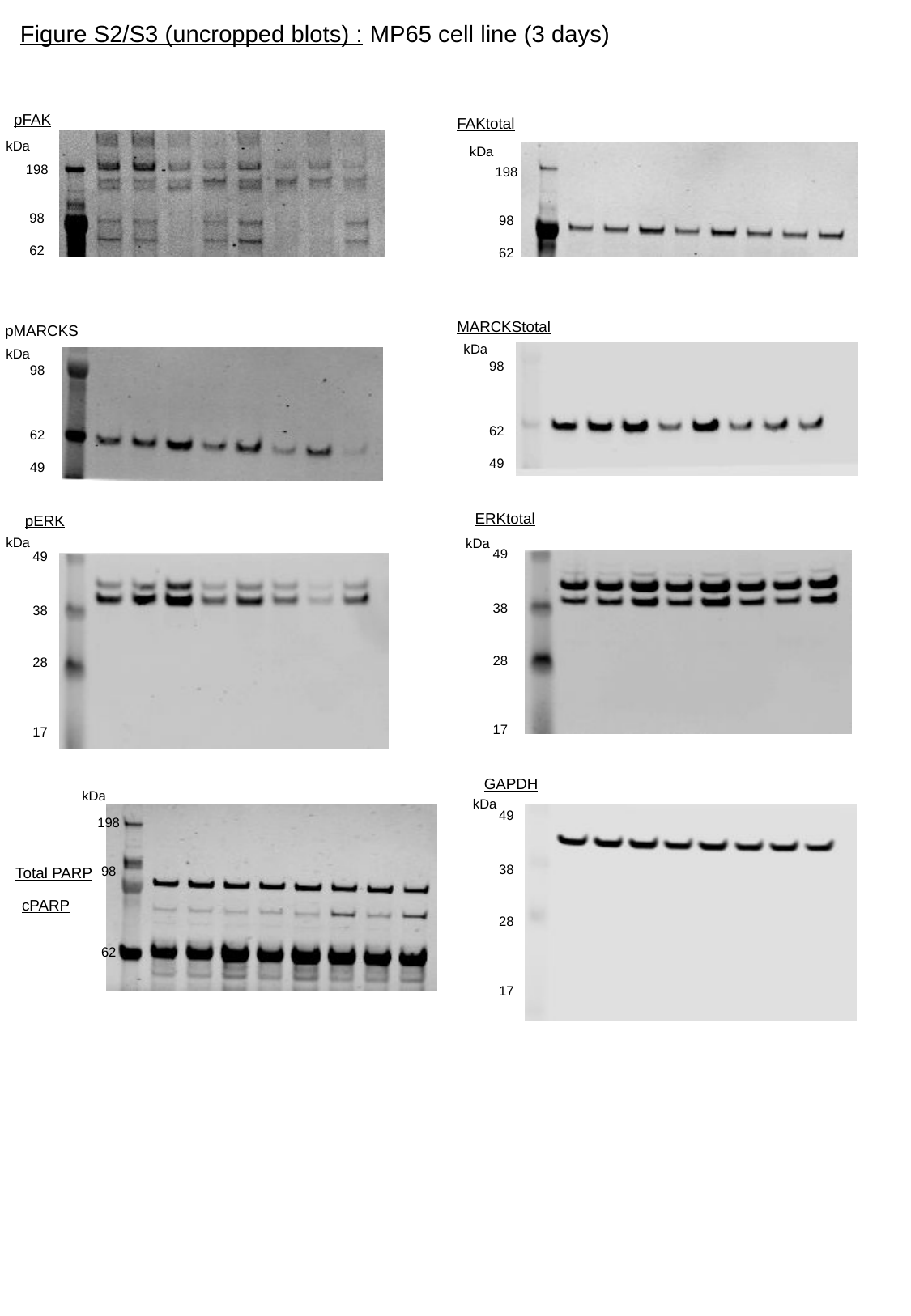

Figure S2/S3 (uncropped blots) : MP65 cell line (3 days)
pFAK
FAKtotal
kDa
kDa
198
98
62
198
98
62
MARCKStotal
pMARCKS
kDa
kDa
98
62
49
98
62
49
ERKtotal
pERK
kDa
kDa
49
38
28
17
49
38
28
17
GAPDH
kDa
kDa
49
38
28
17
198
98
62
Total PARP
cPARP

## Slide 11
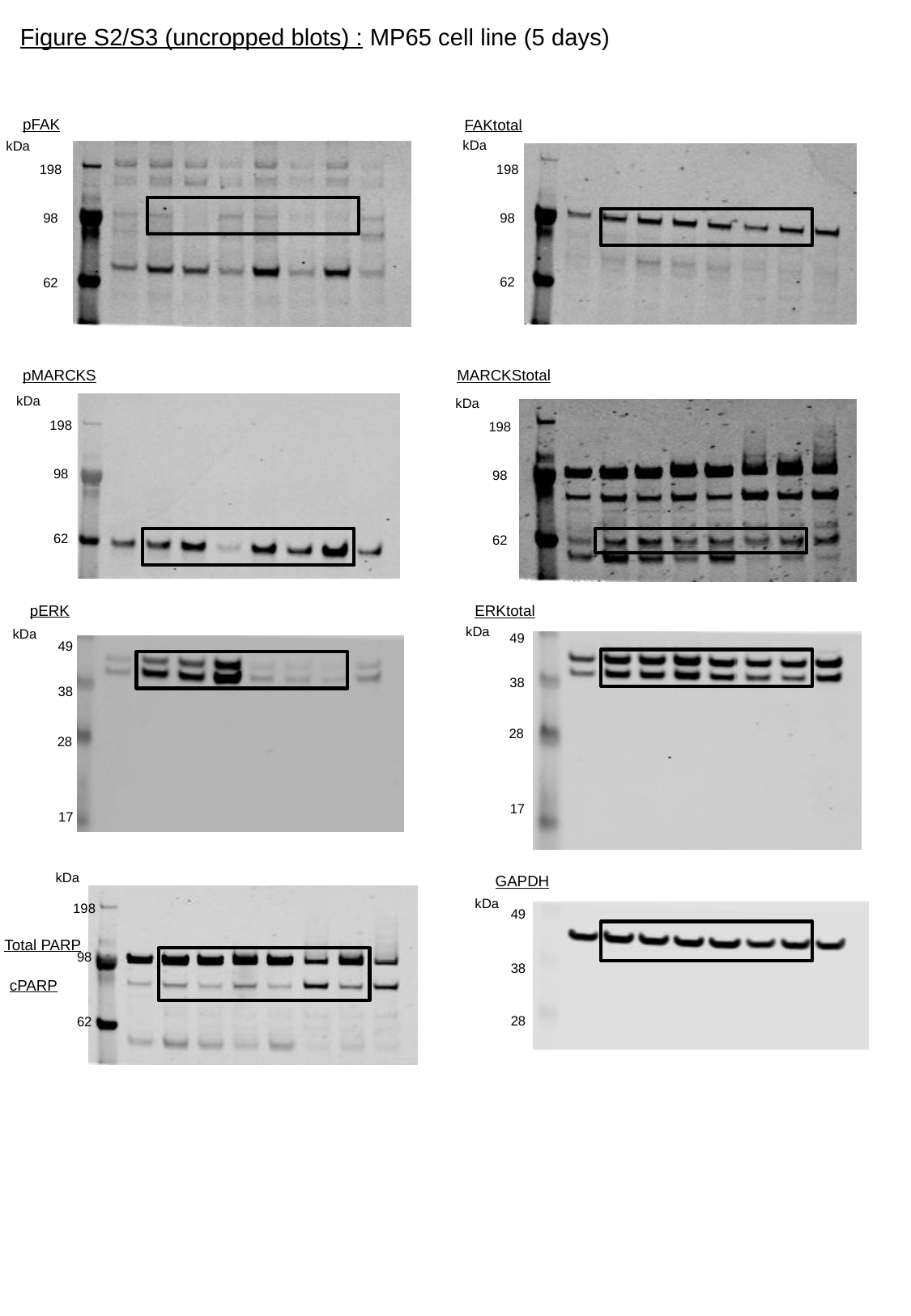

Figure S2/S3 (uncropped blots) : MP65 cell line (5 days)
pFAK
FAKtotal
kDa
kDa
198
98
62
198
98
62
pMARCKS
MARCKStotal
kDa
kDa
198
98
62
198
98
62
pERK
ERKtotal
kDa
kDa
49
38
28
17
49
38
28
17
kDa
GAPDH
kDa
198
98
62
49
38
28
Total PARP
cPARP

## Slide 12
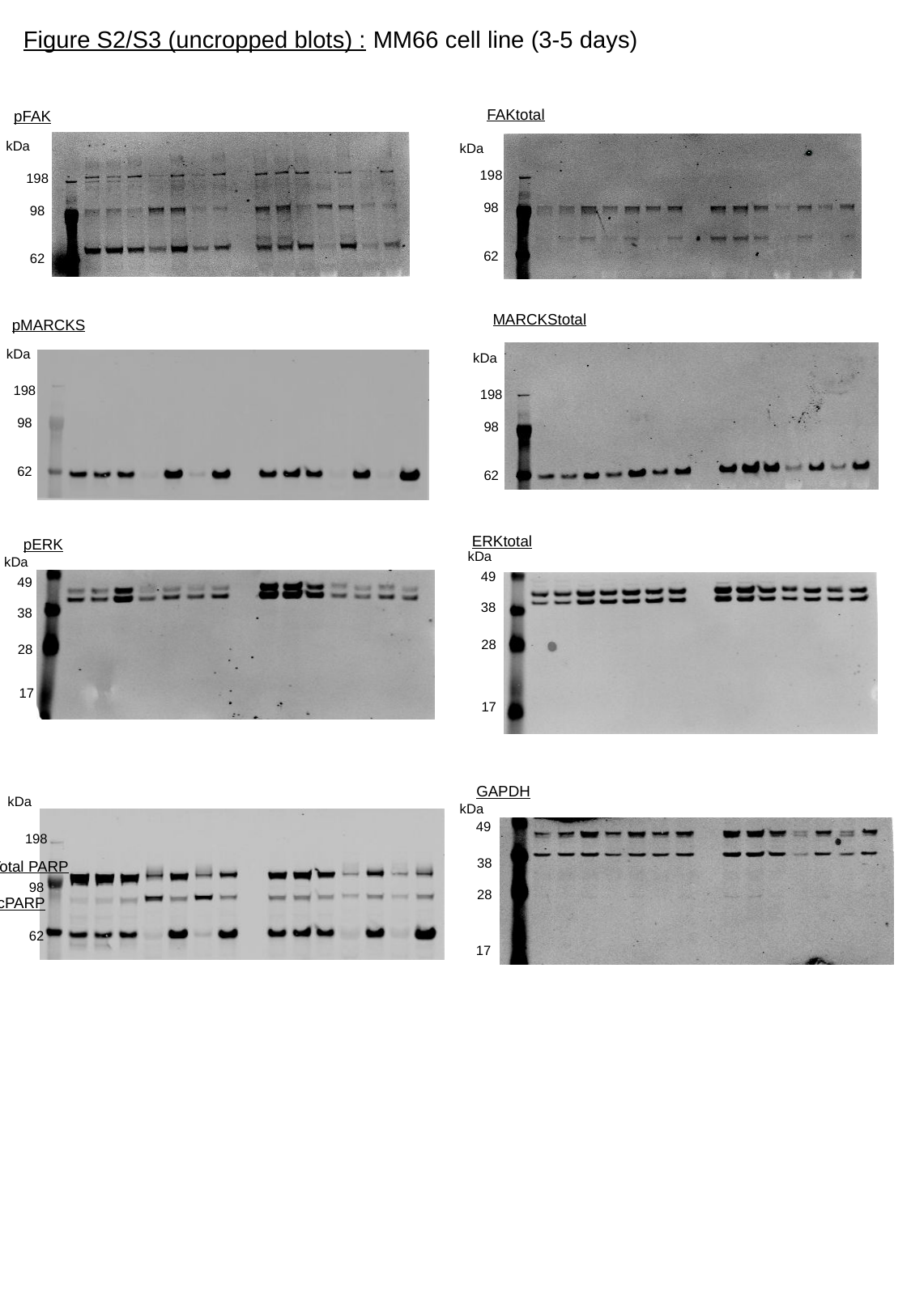

Figure S2/S3 (uncropped blots) : MM66 cell line (3-5 days)
FAKtotal
pFAK
kDa
kDa
198
98
62
198
98
62
MARCKStotal
pMARCKS
kDa
kDa
198
98
62
198
98
62
ERKtotal
pERK
kDa
kDa
49
38
28
17
49
38
28
17
GAPDH
kDa
kDa
49
38
28
17
198
98
62
Total PARP
cPARP

## Slide 13
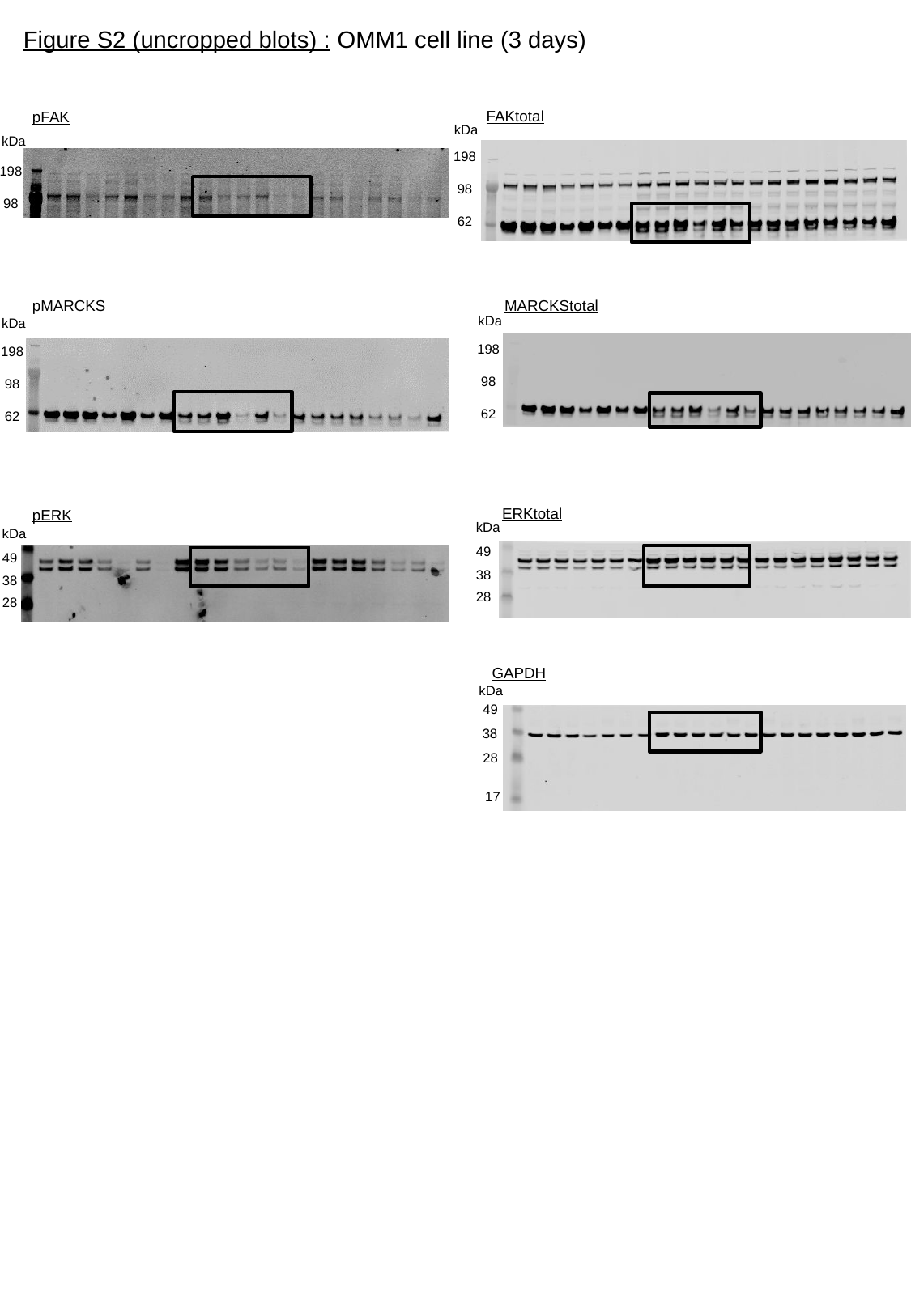

Figure S2 (uncropped blots) : OMM1 cell line (3 days)
FAKtotal
pFAK
kDa
kDa
198
98
62
198
98
pMARCKS
MARCKStotal
kDa
kDa
198
98
62
198
98
62
ERKtotal
pERK
kDa
kDa
49
38
28
49
38
28
GAPDH
kDa
49
38
28
17

## Slide 14
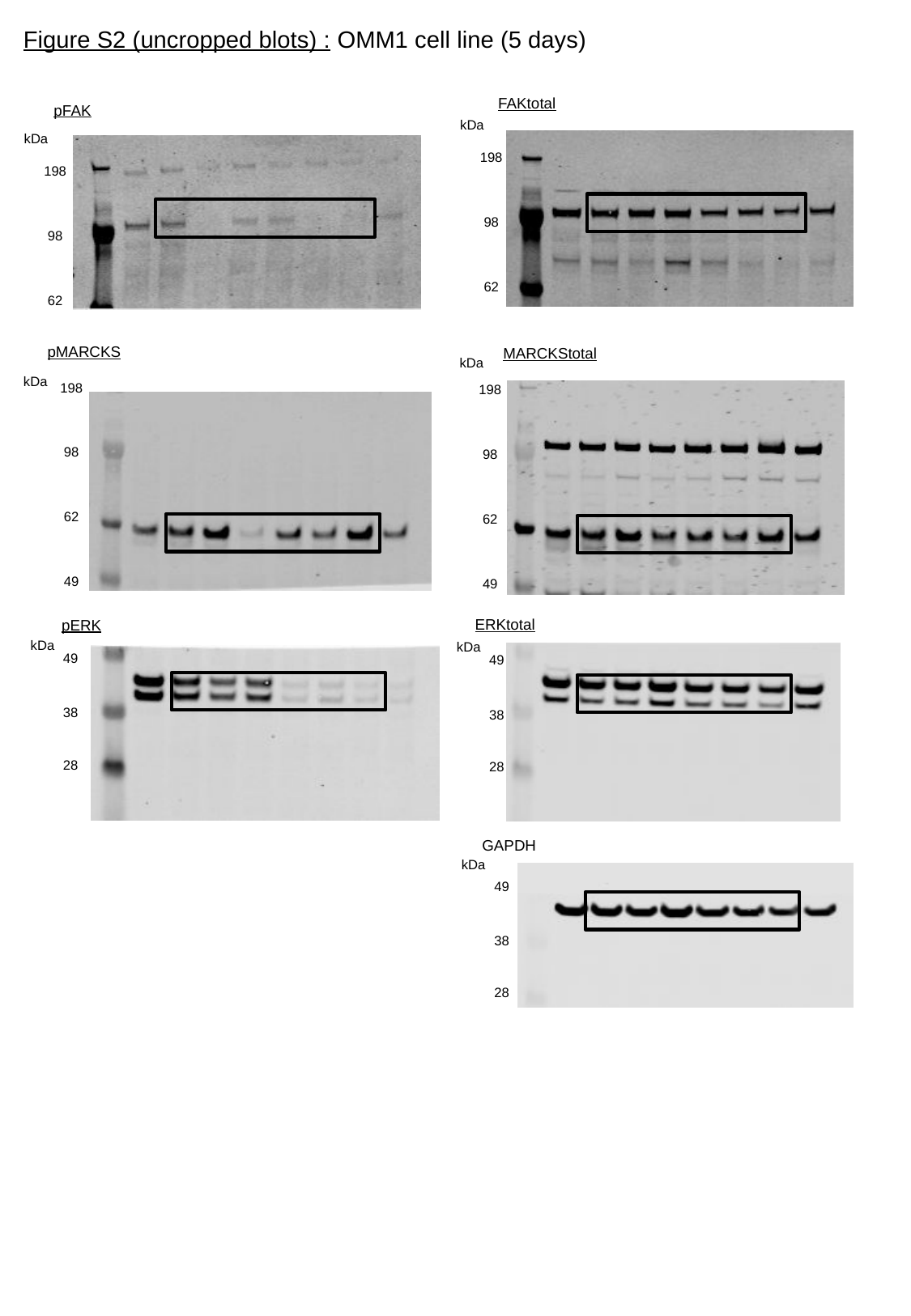

Figure S2 (uncropped blots) : OMM1 cell line (5 days)
FAKtotal
pFAK
kDa
kDa
198
98
62
198
98
62
pMARCKS
MARCKStotal
kDa
kDa
198
98
62
49
198
98
62
49
ERKtotal
pERK
kDa
kDa
49
38
28
49
38
28
GAPDH
kDa
49
38
28

## Slide 15
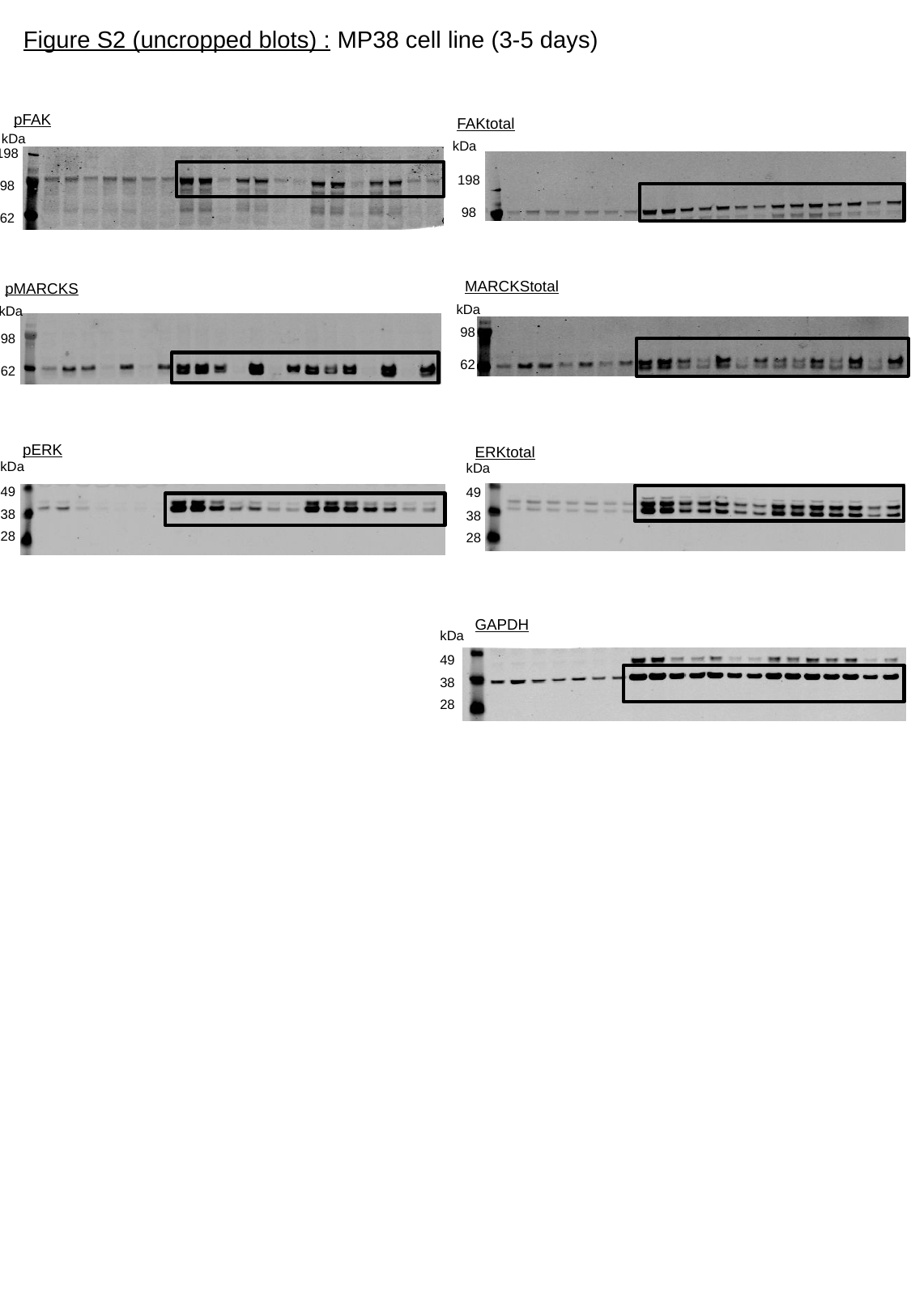

Figure S2 (uncropped blots) : MP38 cell line (3-5 days)
pFAK
FAKtotal
kDa
kDa
198
98
62
198
98
MARCKStotal
pMARCKS
kDa
kDa
98
62
98
62
pERK
ERKtotal
kDa
kDa
49
38
28
49
38
28
GAPDH
kDa
49
38
28

## Slide 16
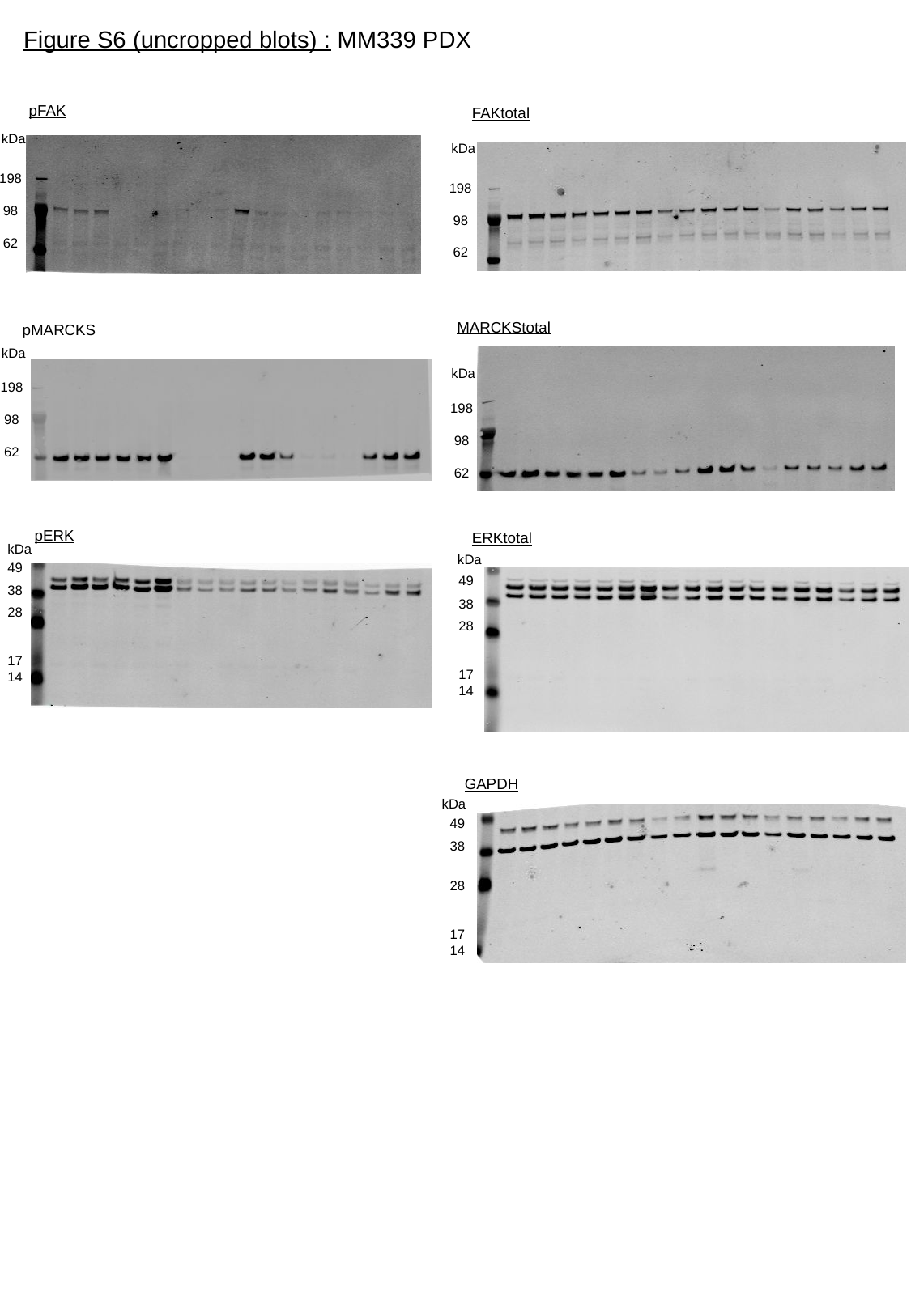

Figure S6 (uncropped blots) : MM339 PDX
pFAK
FAKtotal
kDa
kDa
198
98
62
198
98
62
MARCKStotal
pMARCKS
kDa
kDa
198
98
62
198
98
62
pERK
ERKtotal
kDa
kDa
49
38
28
17
14
49
38
28
17
14
GAPDH
kDa
49
38
28
17
14
