# Supplementary material for: FAK Inhibitor-Based Combinations with MEK or PKC Inhibitors Trigger Synergistic Antitumor Effects in Uveal Melanoma
Source: Cancers (Basel). 2023 Apr 13;15(8):2280. doi: 10.3390/cancers15082280 (PMC10136875; doi:10.3390/cancers15082280)
Supplement: Supplementary file 1 [file cancers-15-02280-s001.zip › REV Supp_Tables.pdf]

Supplementary Table S1. Uveal melanoma cell lines used in the drug combination screen.

		OMM1	OMM2.3	OMM2.5	MM66	MM28	MP38	MP46	MP65
Origin		Sub-cutaneous metastasis	Liver metastasis	Liver metastasis	PDX from liver metastasis	PDX from liver metastasis	Primary tumor	PDX from primary tumor	Primary tumor
Molecular alterations	GNAQ	-	c.626A>C	c.626A>C	-	-	c.626A>T	c.626A>T	-
	GNA11	c.626A>T	-	-	c.626A>T	c.626A>T	-	-	c.626A>T
	BAP1	+	+	+	+	-	-	-	-

Cells were cultured in RPMI-1640 supplemented with 10% fetal bovine serum (FBS) (OMM1, OMM2.3, OMM2.5) or 20% FBS (MP38, MP46, MP65, MM28, MM66). All cells were maintained at 37°C in a humidified atmosphere with 5% CO_2 and were tested and certified as mycoplasma free.

Supplementary Table S2. Targeted pathways and compounds tested in the drug combination screen. Five serial 1:3 dilutions starting from the maximal concentration were evaluated in the screening.

Pathways	Targets	Compounds	Maximal concentration (µM)
PKC	PKC	LXS-196	2
MAPK/ERK	MEK1/2	Trametinib	0,005
FAK/YAP	FAK	VS-4718	2

Supplementary Table S3: Main characteristics of the tested uveal melanoma PDXs.

PDXs	Histology	Monosomy 3	8q gain	*GNAQ* mutation	*GNA11* mutation	*BAP1* mutation	*SF3B1* mutation
MM26	E	+	+	+	0	0	+
MM309	M	+	+	+	0	+	0
MM339	M	+	+	0	+	+	0

Abbreviations: E, epithelioid cells; M, mixed cells.

Supplementary Table S4: Compounds tested in *in vivo* experiments

Compounds	Targets	Drug preparation	Route	Dose per administration (mg/kg)	Schedule of treatment
Trametinib	MEK1/2	DMSO 5% / HMPC 0.5%/ water	PO*	0.4	4 days/week**
LXS-196	PKC	0,5% Tween 80/ 0.5%Methylcellulose / 90%H2O	PO	75$	BID, 5days/week
VS-4718	FAK	5% DMSO / 30%polypropylene glycol/ 40% PEG300 / 25% NaCl 0,9%	PO	60£	BID, 5days/week

*PO, *per os*; $: 150mg/kg/day; £: 120mg/kg/day; **: except for MM339 (5 days/week)

Two different schedules were assessed to evaluate anti-tumor efficacy with an administration four or five days per week every week (continuous schedule) or every two weeks (discontinuous schedule).

Supplementary Table S5: Intensity readings of each band for all immunoblotting. All intensity bands have been quantified with ImageJ

OMM1

	1h						2h					
VS4718	-	+	-	-	+	+	-	+	-	-	+	+
LXS196	-	-	+	-	+	-	-	-	+	-	+	-
Trametinib	-	-	-	+	-	+	-	-	-	+	-	+
pFAK	14764	4217	12217	19111	7246	4589	8929	6393	15124	16581	6444	5616
FAK	19180	23436	18489	20482	20917	19255	16598	22965	22634	18271	18387	18920
pERK	22485	22941	15202	245	7862	248	16205	22121	19304	1047	10047	794
ERK	21769	23675	20751	23773	23443	23315	15166	20607	19653	14325	17244	17174
pMARCKS	17983	19052	6572	19761	7739	19271	18224	20142	11689	19879	9361	19614
MARCKS	21526	22328	14450	20512	12599	17823	14813	19928	19804	17886	12737	17510
GAPDH	21640	24431	22574	22263	20588	22354	20662	25972	24486	21054	21594	21392

	3 days						5 days					
VS4718	-	+	-	-	+	+	-	+	-	-	+	+
LXS196	-	-	+	-	+	-	-	-	+	-	+	-
Trametinib	-	-	-	+	-	+	-	-	-	+	-	+
pFAK	11017	2847	8878	7403	2637	2019	10842	832	3758	5739	1696	2607
FAK	6963	9896	5836	9325	4085	4556	12596	13392	13714	9039	8618	7255
pERK	9490	16197	8279	4073	2409	1410	12860	8107	9649	1228	1662	878
ERK	10555	13103	13751	18949	11201	9534	12512	12171	15969	11778	10564	8135
pMARCKS	9912	14272	4436	13448	2197	13951	10306	13588	1533	8106	6252	13194
MARCKS	11026	13738	8521	14813	8277	13623	15188	14552	11529	11977	10536	13037
cPARP	9457	5974	4980	6098	7406	4543	8277	9532	7909	8939	11796	13097
PARP	12946	11212	12897	13035	7428	5019	11109	10983	10205	12682	12700	16318
GAPDH	16176	15276	15270	18809	12527	11477	15711	14469	17658	14969	13915	11659

MP65

	1h						2h					
VS4718	-	+	-	-	+	+	-	+	-	-	+	+
LXS196	-	-	+	-	+	-	-	-	+	-	+	-
Trametinib	-	-	-	+	-	+	-	-	-	+	-	+
pFAK	27755	9710	19828	23711	6259	7550	18408	9322	17638	18499	3175	6789
FAK	12757	14328	16978	15906	18738	16757	12785	18513	19427	14582	11263	12277
pERK	19317	22062	6669	1076	2156	163	7676	11940	4138	813	1233	1314
ERK	13211	20965	22383	19989	22628	20278	15957	20200	22744	19387	11256	14040
pMARCKS	15371	18338	5942	21562	5924	17464	12517	15289	4080	15608	1997	17588
MARCKS	15874	19279	8071	16457	10412	18287	8686	11287	5567	18411	7143	19262
GAPDH	16688	20751	24812	20110	23133	21244	15385	20621	19526	15187	7901	12656

	3 days						5 days					
VS4718	-	+	-	-	+	+	-	+	-	-	+	+
LXS196	-	-	+	-	+	-	-	-	+	-	+	-
Trametinib	-	-	-	+	-	+	-	-	-	+	-	+
pFAK	9884	2586	7880	9636	3430	3388	11370	4341	7779	10349	3320	4838
FAK	9110	13312	7763	12135	8919	7644	9646	10519	9922	9177	5215	7956
pERK	11374	17001	6628	10853	6480	1631	10164	11212	17468	1532	1237	745
ERK	10668	14436	10707	16164	11036	10153	13748	13565	16271	12459	9646	8738
pMARCKS	10710	13675	6785	11483	3660	8968	7730	10097	1655	10129	6702	13129
MARCKS	13213	15050	6649	14966	7123	9458	10516	10180	7389	8756	7990	8836
cPARP	3052	3631	3936	3969	8949	4836	6275	3450	8649	3295	13382	6417
PARP	12809	12112	12151	12324	11248	11581	14881	13456	15020	13498	8696	13635
GAPDH	16696	17375	14336	16024	13976	14454	16976	16071	15788	16161	11622	13535

MP38

	1h						2h					
VS4718	-	+	-	-	+	+	-	+	-	-	+	+
LXS196	-	-	+	-	+	-	-	-	+	-	+	-
Trametinib	-	-	-	+	-	+	-	-	-	+	-	+
pFAK	16663	3897	17998	17419	6287	2811	13762	2322	13386	21758	3625	2808
FAK	16862	17304	17261	17449	20634	20551	15700	14410	13820	19683	18107	19807
pERK	20872	22767	10746	924	9709	912	18066	19588	5717	1778	10435	1321
ERK	16374	19739	15297	15592	21405	21716	16915	19708	19653	24080	23808	22674
pMARCKS	18921	19454	2218	18179	3508	21039	17404	21082	1764	20157	1932	19260
MARCKS	18392	18320	6488	16702	7742	18273	17632	20121	7014	19482	5347	19688
GAPDH	16947	22071	16712	17068	18669	18852	16229	15819	16609	18762	18431	19715

	3 days						5 days					
VS4718	-	+	-	-	+	+	-	+	-	-	+	+
LXS196	-	-	+	-	+	-	-	-	+	-	+	-
Trametinib	-	-	-	+	-	+	-	-	-	+	-	+
pFAK	7809	1252	4440	5342	1318	836	6425	1656	4571	5394	2405	3010
FAK	19782	11843	8621	11365	8123	7090	13738	16412	10108	13252	6609	12126
pERK	9900	6042	2138	2750	1105	815	8406	7577	4747	3953	1254	1487
ERK	8612	3737	4145	5511	2584	2113	5618	7282	5610	6053	1815	3999
pMARCKS	7780	4020	167	9751	510	5028	3487	6367	292	9783	269	8521
MARCKS	8924	4526	2344	8508	1474	4804	3323	6722	4045	9108	2590	8016
cPARP	7959	3743	3104	5469	3295	2808	4692	5971	3159	5089	3410	5820
PARP	8984	4497	4555	5797	3717	2584	6038	7993	4843	5330	2717	4332
GAPDH	10260	6990	6617	7762	4895	4717	7892	8641	6725	7133	2927	6172

MM66

	1h						2h					
VS4718	-	+	-	-	+	+	-	+	-	-	+	+
LXS196	-	-	+	-	+	-	-	-	+	-	+	-
Trametinib	-	-	-	+	-	+	-	-	-	+	-	+
pFAK	17723	5977	15054	18064	5129	4701	17723	5977	15054	18064	5129	4701
FAK	13139	21103	14851	11119	17678	14747	11756	14330	10218	13913	19170	17777
pERK	21038	15990	5762	1294	3764	725	19078	16633	2730	2033	2852	609
ERK	12766	24649	16473	16898	21912	19880	16452	20971	14027	17040	20330	18919
pMARCKS	11934	22674	314	16452	855	17488	13748	17646	55	13743	339	15333
MARCKS	13013	21731	11407	16634	17289	17782	11717	15215	3957	15230	12988	18843
GAPDH	13572	20559	16028	12686	17458	18607	18427	20705	20512	21421	22645	20772

	3 days						5 days					
VS4718	-	+	-	-	+	+	-	+	-	-	+	+
LXS196	-	-	+	-	+	-	-	-	+	-	+	-
Trametinib	-	-	-	+	-	+	-	-	-	+	-	+
pFAK	2289	1485	3372	3890	1268	926	3474	1374	2299	2577	1340	2000
FAK	2692	5709	2616	4227	2642	4241	4122	3230	902	3114	1537	3679
pERK	1767	6432	1233	1646	1238	1485	8162	4468	1367	1178	1667	1423
ERK	3195	6337	5413	6128	5594	5311	7520	5438	3703	5293	3764	4998
pMARCKS	4137	4975	263	7913	434	7365	5766	5419	347	7071	395	9814
MARCKS	2154	4781	2214	5557	2468	4436	6799	4960	1041	3924	897	4579
cPARP	1910	2541	7989	3710	8026	3256	4039	2603	1337	2817	1282	3713
PARP	8291	6937	3927	5797	2847	3919	6094	4923	1497	4455	1276	3332
GAPDH	4623	6015	3860	4954	4244	5489	7741	5548	1677	4189	3355	4375

MM339 PDX

	Control			VS4718 + LXS196			VS4718 + Trametinib		
Mice (triplicate)	R1	R2	R3	R1	R2	R3	R1	R2	R3
pFAK	7119	6900	7361	805	2355	1652	441	1557	524
FAK	13799	14975	14080	4127	9937	9258	6355	8351	8807
pERK	8624	13316	9403	2061	3358	2685	1493	3197	3817
ERK	13538	15438	14502	11663	14025	15090	7728	12890	12640
pMARCKS	14392	15115	13989	608	1001	399	10578	15445	15643
MARCKS	11749	15467	11916	2098	7984	7859	5835	10964	10883
GAPDH	17159	16703	16260	10378	14600	13339	11493	12768	13526
